# Synergistic Regulation of Nitrogen and Sulfur on Redox Balance of Maize Leaves and Amino Acids Balance of Grains

**DOI:** 10.3389/fpls.2020.576718

**Published:** 2020-12-04

**Authors:** Shuoran Liu, Shuai Cui, Xue Zhang, Yin Wang, Guohua Mi, Qiang Gao

**Affiliations:** ^1^Key Laboratory of Sustainable Utilization of Soil Resources in The Commodity Grain Bases of Jilin Province, College of Resource and Environmental Sciences, Jilin Agricultural University, Changchun, China; ^2^College of Resources and Environmental Science, China Agricultural University, Beijing, China

**Keywords:** cysteine, glutathione, maize, nitrogen, photosynthesis, Rubisco, sulfur

## Abstract

As a primary food crop, maize is widely grown around the world. However, the deficiency of essential amino acids, such as lysine, tryptophan, and methionine, results in poor nutritional quality of maize. In addition, the protein concentration of maize declines with the increase in yield, which further reduces the nutritional quality. Here, the photosynthesis of leaves, grain amino acid composition, and stoichiometry of N and S are explored. The results show that N and S maintained the redox balance by increasing the content of glutathione in maize leaves, thereby enhancing the photosynthetic rate and maize yield. Simultaneously, the synergy of N and S increased the grain protein concentration and promoted amino acid balance by increasing the cysteine concentration in maize grains. The maize yield, grain protein concentration, and concentration of essential amino acids, such as lysine, tryptophan, and methionine, could be simultaneously increased in the N:S ratio range of 11.0 to 12.0. Overall, the synergy of N and S simultaneously improved the maize yield and nutritional quality by regulating the redox balance of maize leaves and the amino acids balance of grains, which provides a new theoretical basis and practical method for sustainable production of maize.

## Introduction

Contemporary grain production faces great challenges, including that more than 1 in 10 people still do not have access to sufficient energy and protein in their diets even with recent productivity gains (Food and Agriculture Organization of the United Nations, 2018). As one of the main food crops, maize (*Zea mays* L.) provides 20% of the calories and 15% of the protein in the global diet, making an important contribution to global food security (Bhatnagar et al., 2004). In terms of production, maize has become the most productive cereal crop (Liu et al., 2020). However, the serious deficiency of essential amino acids (EAA), such as lysine, tryptophan, and methionine, results in an amino acid imbalance in the grain, which often requires expensive dietary supplementation (Ali et al., 2011; Li et al., 2020). Simultaneously, in maize production, a yield increase usually results in a continuous decline in protein concentration, which decreases by an average of 0.3% per decade (Duvick, 2005; Chen et al., 2015). One study based on 45 maize varieties from the 1920s through 2001 shows that the increase in maize yield was mainly achieved by enhancing the starch concentration in the grains. The concentration of EAA, such as lysine, tryptophan, and methionine, in maize grains decreased as the yield increased (Scott et al., 2006). In addition, climate change also seriously affects food yield and nutritional quality (Li et al., 2009; Soares et al., 2019). Therefore, how to synchronously improve grain yield and nutritional quality is a great challenge in maize production.

Among the many strategies to increase crop yield, increasing the efficiency and productivity of photosynthesis is widely accepted as the pivotal measure (Leister, 2012; Long et al., 2015; Foyer et al., 2017; Wu et al., 2019). However, because it is a key catalytic enzyme for photosynthesis, the low efficiency of ribose-1,5-bisphosphate carboxylase/oxygenase (Rubisco) has always plagued attempts at the improvement of photosynthesis (Igamberdiev, 2015; Sharwood, 2016). Consequently, plants need a significant nitrogen (N) investment to synthesize a large amount of Rubisco for carbohydrate synthesis (Whitney and Sharwood, 2014). In fact, Rubisco is the most abundant protein in plants, accounting for 50% of the soluble protein in the leaves, and 25% of the N in the leaves is used to synthesize Rubisco (Parry et al., 2013). It has been suggested that N deficiency causes concentration of Rubisco to be significantly reduced in leaves, which leads to decreased rates of chloroplast electron transport (Osaki et al., 1993; von Caemmerer et al., 2004). Furthermore, N deficiency severely reduces the maximum carboxylation rate of Rubisco, the photosynthetic rate, and the use of triose-P parameters (Rubio-Wilhelmi et al., 2014). Similarly, as an important component of Rubisco, sulfur (S) also affects its metabolism and activity (Ashida et al., 2005). In plants of S deficiency, the levels of chlorophyll and the Rubisco in leaves are reduced twofold and sixfold, respectively, and PSII efficiency is reduced by 31%, which results in a significant reduction in photosynthesis efficiency (Lunde et al., 2008). In addition, some studies show that the deficiency of N and S can reduce the intensity of photosynthesis by affecting the content of hydrogen peroxide (H_2_O_2_) and glutathione (GSH) in maize plants (Bashir et al., 2020; Nemat Alla and Hassan, 2020). In conclusion, the deficiency of N and S inhibits the photosynthesis of crops and ultimately leads to yield reduction (Resurreccion et al., 2002; Ding et al., 2005).

In terms of the nutritional quality of maize, it is thought that increasing the protein concentration of grains could improve the nutritional quality of maize. However, an imbalance of amino acids in the diet could cause serious negative effects (Maurin et al., 2014). Therefore, in order to obtain balanced nutrition, it is not enough to consider the accumulation of protein; the proportion of amino acids in dietary protein should also be considered (Kim et al., 2019). Studies show that simply increasing the protein concentration of maize grains may not necessarily improve its nutritional quality and even has a negative impact (Wu and Messing, 2012). MacGregor et al. (1961) found that N application could significantly increase the protein concentration of maize grains, but the concentration of each amino acid in the protein did not increase uniformly. Among all amino acids, the concentration of non-essential amino acids (NAA), such as glutamic acid and proline, continuously increase with the increase of N application rates, and EAA, such as lysine and methionine, continuously decrease with the increase of N application rates. Tsai et al. (1983) reported that, as the N application rates increased, zein accumulated preferentially in maize grains, and the concentration of lysine and tryptophan continuously decreased as the protein concentration increased. Subsequent studies show that, with the N application rates increased, the zeins lacking EAA significantly increased, and the concentration of EAA, such as lysine and threonine, continuously decreased, which exacerbated the imbalance of amino acids in grains (Tsai et al., 1992; Lošák et al., 2010).

Contrary to N, S can increase the concentration of EAA in maize grains, especially the concentration of sulfur-containing amino acids, such as cysteine and methionine (Habtegebrial and Singh, 2009). Many studies show that the nutritional quality of crops can be improved by increasing the concentration of methionine and cysteine in crops (Galili and Amir, 2013; Krishnan and Jez, 2018). It is argued that enhanced S storage can increase the concentration of methionine and cysteine in maize grains, thereby promoting the balance of amino acids in grains (Wu et al., 2012; Planta et al., 2017). In fact, the concentration of methionine and cysteine in S-deficient maize grains decreased by 25% and 30%, respectively, and the concentration of asparagine and aspartic acid increased by 30%, which seriously reduced the nutritional quality of maize (Baudet et al., 1986). It can be seen that the regulation effects of N and S on the amino acids in maize grains are different. It is worth optimizing the amino acid balance of maize grains by coordinating the supply of N and S.

As essential mineral nutrient elements for proteins, enzymes, coenzymes, prosthetic groups, vitamins, amino acids, GSH, and secondary metabolites, N and S have important regulatory effects on crop growth, yield, and nutritional quality (Hawkesford et al., 2012; Gigolashvili and Kopriva, 2014). In actual agricultural production, N and S not only independently exert their functions, but also interact with each other. Some studies show that the application rates of N and S and the stoichiometry of N and S in maize plants have an impact on maize yield and nutrient use efficiency (Li et al., 2019; Carciochi et al., 2020). However, synergistically improving maize yield and nutritional quality by regulation of N and S is seldom reported. Therefore, a pot experiment combining N and S fertilization was established to investigate the regulation mechanism of N and S for synergistically improving maize yield and grain protein quality. The relationship between yield, protein concentration, amino acid composition of grains and stoichiometry of N and S in grains was analyzed in this experiment. Besides that, the response of the GSH content, H_2_O_2_ content, Rubisco activity, photosynthetic rate, and concentration of N and S in leaves to application rates of N and S was also investigated at important growth stages of maize. In this study, the regulation mechanism of N and S was proposed to synergistically enhance maize yield and nutritional quality by maintaining the redox balance in maize leaves and promoting amino acid balance in maize grains.

## Materials and Methods

### Experimental Design

The experiment was undertaken in a greenhouse at the experimental base of Jilin Agricultural University in 2017 and 2018. The greenhouse temperature was the same as the outdoor temperature. During this experiment, soil samples used for the test were sandy soils with the following characteristics: pH 5.77 (1:2.5 m/v), soil organic matter (SOM) 16.2 g kg^–1^, available nitrogen (alkali-hydrolyzable N) 61.71 mg kg^–1^, available phosphorus (Olsen-P) 24.96 mg kg^–1^, available potassium (1 mol L^–1^ NH_4_OAc extracting) 120.92 mg kg^–1^, and available sulfur [0.008 mol L^–1^ Ca(H_2_PO_4_)_2_ extracting] 11.36 mg kg^–1^. Maize (cv. Liangyu 99) seeds were planted in plastic pots (Φ × h = 30 cm × 35 cm) filled with an equal quantity of soil (25 kg pot^–1^). In each pot, the same amount of water (70%–75% of the maximum water-holding capacity of the soil) was maintained, and the weight of water was controlled using a weighing method. The experiment was a two-factor interaction design of different N and S fertilization levels. Sixteen treatments of a combination of N and S and four repetitions for each treatment were undertaken. Four rates of N, i.e., no N (N0), low N (N1), moderate N (N2), and high N (N3) were applied. The amount of fertilization (g fertilizer kg soil^–1^) was 0, 0.06, 0.24, and 0.48 g kg^–1^. Four rates of S, i.e., no S (S0), low S (S1), moderate S (S2), and high S (S3) were applied. The amount of fertilization was 0, 0.04, 0.12, and 0.24 g kg^–1^. The fertilizers used for the test were all AR grade. The types were as follows: nitrogen (N) [CO(NH_2_)_2_, N = 46.2%], phosphorus (P) (KH_2_PO_4_, P_2_O_5_ = 52.1%, K_2_O = 34.6%), potassium (K) (KCl, K_2_O = 62.5%), and sulfur (S) (MgSO_4_⋅7H_2_O, S = 13.0%). The same amount of P (P_2_O_5_ = 0.15 g kg^–1^) and K (K_2_O = 0.15 g kg^–1^) fertilizers were maintained in each pot. In the course of filling the pots with soil, total N of the N1 treatment; 1/3 N of the N2 and N3 treatments; and total P, K, and S were applied in every pot as a basal fertilizer mixed with the soil. The remaining 2/3 N of the N2 and N3 treatments was used a topdressing fertilizer in the corresponding pots at the stage when the maize unfolds the eighth leaf. Initially, five maize seeds per pot were planted to a depth of 2 cm. When the seedlings had grown six leaves, one representative seedling was reserved, and the remaining seedlings were removed from the pots. During the entire plant’s growth, conventional management for prevention and control of pests and plant diseases was conducted.

### Photosynthetic Rate Measurements

The photosynthetic rate of maize leaves (the first leaf above the ear of the maize plant in each experimental treatment) was measured by a Li-6400XT portable photosynthesis system (Li-6400XT, Li-Cor, Inc., Lincoln, NE, United States) at the silking stage and the grain-filling stage (75 and 100 days after planting, DAP), respectively. The measurements were performed during the morning of a sunny day (9:00–11:00). The leaf chamber light intensity of the head light source was set to 1600 μmol m^–2^ s^–1^ photosynthetic photon flux density (PPFD).

### H_2_O_2_ Content, GSH Content, and Rubisco Activity Measurements

The collection of samples for biochemical measurement was performed simultaneously with the determination of photosynthesis. The 10-cm^2^ leaf disks collected from the first leaf above the maize ear were quickly placed in liquid nitrogen and stored in a refrigerator at −80°C until extraction (Perdomo et al., 2017). According to the relevant measurement method (Pan et al., 2017; Hou et al., 2018), the H_2_O_2_ content, GSH content, and Rubisco activity of maize leaves were determined by an enzyme-linked immunosorbent assay method with commercial kits (Jiangsu Kete Biological Science and Technology Co., Ltd., China).

### Maize Yield Measurements

At the maturity stage (140 DAP), the plant was cut along the soil surface, and the grains were threshed. All maize grains from each plant were collected and placed in a constant temperature oven at 70°C to dry to a constant weight and weighed.

### Grain Ultrastructure Analysis

The grain samples were placed overnight in 2% (v/v) glutaraldehyde in a 0.1 mol L^–1^ phosphate buffer (Buffer A) with a pH of 7.2. The samples were rinsed three times in buffer A for 5 min each time and dehydrated with a gradient series of ethanol (25–100%). The maize grains were horizontally cut into slices (1 mm) and dried in a lyophilizer. The prepared samples were observed and photographed using a scanning electron microscope (SEM) (SU8000, HITACHI, Japan).

### Concentration and Stoichiometry of N and S Measurements

At the silking (75 DAP), grain-filling (100 DAP), and maturity stages (140 DAP), a portion (about 50 g, fresh weight, FW) of the leaf near the maize ear was cut and dried in a constant temperature oven at 70°C and then ground into powder for measuring the N and S concentration. Similarly, the maize grains at maturity were dried and ground to measure the N and S concentration. The samples were digested with acid (H_2_SO_4_-H_2_O_2_), cooled to room temperature, and equilibrated with deionized water. Then, the N concentration was measured by a Kjeldahl instrument (KDY-9820, KETUO, China). After the samples were digested with acid (HNO_3_-HClO_4_), the S concentration was measured using an inductively coupled plasma instrument (SHIMADZU, I-7500, Japan). The stoichiometry of N and S was determined by the ratio of N to S concentration in the samples.

### Grain Protein Concentration Measurements

The concentration of protein in maize grains (*P*_*c*_) was converted from the N concentration of grains (*N*_*c*_) measured by the Kjeldahl method using the following equation:

*P*_*c*_ = *N*_*c*_ × 6.25

### Amino Acid Analysis

An appropriate amount (0.05 g) of the sample was placed in a 20-ml hydrolysis tube, and 20 ml of 6 mol L^–1^ HCl was added. The tube was sealed with nitrogen and hydrolyzed at 110°C for 24 h. The sample used for the determination of tryptophan was hydrolyzed with 5 mol L^–1^ NaOH for 24 h, and the pH of the solution was adjusted to 6 with 6 mol L^–1^ HCl. The amino acid analysis was carried out using a high-performance liquid chromatography instrument (1260 Infinity II, Agilent, United States).

### Statistical Analysis

The statistically experimental data were compared using two-way analysis of variance (ANOVA). The least significant difference (LSD) test was used to compare significant differences based on *P* values < 0.05. Statistical computations and analysis were conducted using the Statistical Analysis System (SAS 9.2, SAS Institute Inc., United States).

## Results

### Physiological Response in Photosynthesis

**FIGURE 1:**
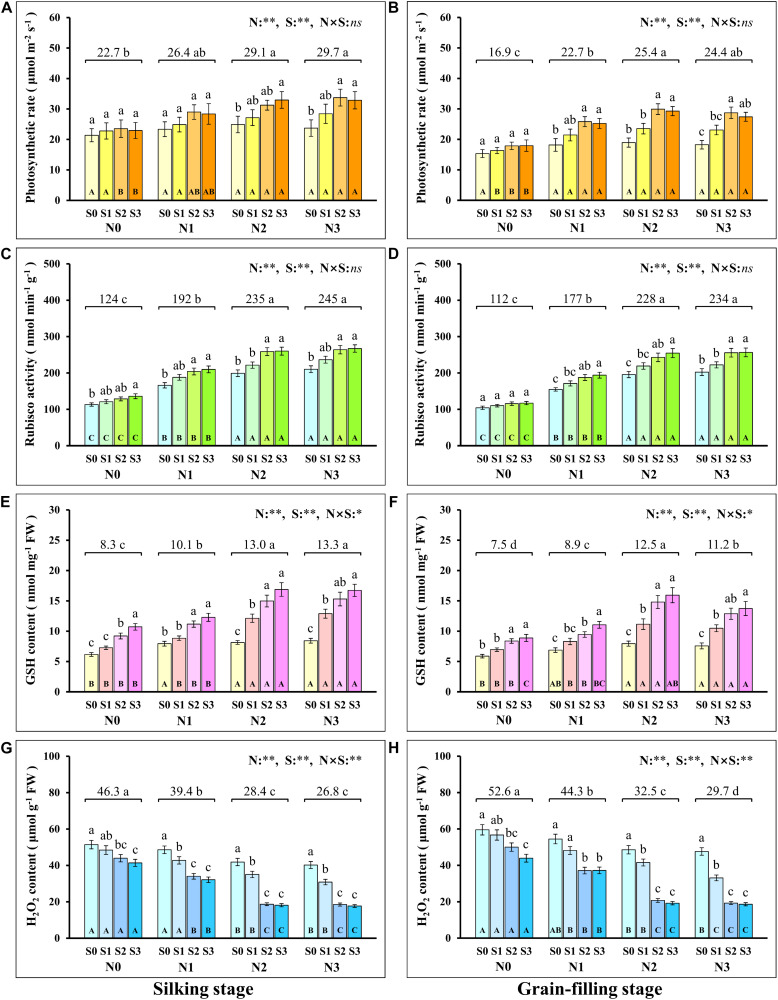
Physiological response during photosynthesis at different N and S rates. The data are the means of four replicates, and the error bars represent the standard deviations. The lowercase letters above the bars indicate the significant differences between the different S rates at the same N level, and the different uppercase letters in the bars indicate the significant differences between the different N rates at the same S level. The number on the horizontal line above each group of bars indicates the average value of the corresponding indicator represented by the ordinate axis at different N levels, and the lowercase letters after the number indicate that the indicator has significant differences at different N levels. The variance analysis used two-way ANOVA (^∗∗^*P* < 0.01, ^∗^*P* < 0.05, *ns P* > 0.05). Response of photosynthetic rate **(A,B)**, Rubisco activity **(C,D)**, GSH **(E,F)**, H_2_O_2_ content **(G,H)** in silking and grain-filling stage to different N and S rates.

During the silking and grain-filling stages of the maize plant, N and S application had significant effects on the concentration of N and S in leaves (Table 1), photosynthetic rate (Figures 1A,B), and Rubisco activity (Figures 1C,D) although their interactions were not significant. Simultaneously, N and S application markedly affected the GSH (Figures 1E,F) and H_2_O_2_ content (Figures 1G,H), and their interaction reached a significant level. During the silking stage, the photosynthetic rate (Figure 1A), Rubisco activity (Figure 1C), and GSH content (Figure 1E) of the leaves increased with N application rates. At the N2 and N3 levels, the photosynthetic rate increased with the S application rate and reached a maximum (33.0 μmol m^–2^ s^–1^) at the N2S3 treatment, which was significantly higher than the N0S0 treatment (21.4 μmol m^–2^ s^–1^). For each N level, the Rubisco activity and GSH content increased with the increase of S application rates and reached a maximum (267.2 nmol min^–1^ g^–1^, 16.9 nmol mg^–1^) at the N3S3 and N2S3 treatments, which was significantly higher than the N0S0 treatment (113.2 nmol min^–1^ g^–1^, 6.2 nmol mg^–1^). During the grain-filling stage, the photosynthetic rate (Figure 1B) and Rubisco activity (Figure 1D) increased with N application rates. Except for the N0 level, the photosynthetic rate and Rubisco activity at other N levels increased with S application rates and reached a maximum (29.9 μmol m^–2^ s^–1^, 256.6 nmol min^–1^ g^–1^) at the N2S2 and N3S3 treatments, respectively. During the grain-filling stage, the GSH content (Figure 1F) increased with N application rates, reached a maximum at the N2 level, and then decreased at the N3 level. For each N level, the GSH content increased with the S application and reached a maximum (15.9 nmol mg^–1^) at the N2S3 treatment. During the silking and the grain-filling, the H_2_O_2_ content (Figures 1G,H) continuously decreased with the increase of S application rates at each N level. For each S level, the H_2_O_2_ content continuously decreased with the increase of N application rates and reached a maximum (17.7 nmol g^–1^, 18.6 nmol g^–1^) at the N3S3 treatment, which has no significant difference compared with the N2S2, N2S3, and N3S2 treatments, but was significantly lower than the N0S0 treatment (51.4 nmol g^–1^, 59.6 nmol g^–1^). Overall, the photosynthetic rate was directly proportional to Rubisco activity and GSH content and inversely proportional to H_2_O_2_ content.

**TABLE 1:** N and S stoichiometry and photosynthetic rate in maize leaves of different growth stages.

Treatment		Silking stage (75 DAP)				Grain-filling stage (100 DAP)			
		*N*_*c*_	*S*_*c*_	N:S	Pn	*N*_*c*_	*S*_*c*_	N:S	Pn
		
		(mg g^–1^)	(mg g^–1^)		(μ mol m^–2^ s^–1^)	(mg g^–1^)	(mg g^–1^)		(μ mol m^–2^ s^–1^)
N0	S0	22.2 a D	1.51 b A	14.8 a C	21.4 a A	16.9 a D	1.35 b A	12.8 a C	15.3 a A
	S1	21.9 a D	1.65 ab A	13.4 a C	22.8 a A	17.0 a D	1.47 ab A	11.8 a C	16.3 a B
	S2	21.4 a C	1.74 ab B	12.4 a C	23.5 a B	16.5 a C	1.56 ab A	10.8 a C	17.9 a B
	S3	20.8 a D	1.80 a B	11.7 a C	22.9 a B	15.9 a D	1.61 a A	10.0 a C	17.9 a B
N1	S0	27.9 a C	1.59 b A	17.8 a BC	23.4 a A	22.0 a C	1.39 b A	16.0 a BC	18.2 b A
	S1	29.5 a C	1.71 ab A	17.4 a B	24.9 a A	23.6 a C	1.52 ab A	15.7 a BC	21.4 ab A
	S2	30.6 a B	1.93 a AB	16.0 a BC	29.0 a AB	24.2 a B	1.68 a A	14.6 a BC	25.8 a A
	S3	30.2 a C	1.98 a AB	15.4 a B	28.4 a AB	23.4 a C	1.74 a A	13.6 a BC	25.2 a A
N2	S0	33.5 b B	1.65 b A	20.5 a AB	24.9 b A	26.3 b B	1.43 b A	18.6 a AB	18.9 b A
	S1	35.7 ab B	1.87 b A	19.3 ab AB	27.1 ab A	28.3 ab B	1.63 ab A	17.7 a AB	23.5 b A
	S2	37.7 a A	2.16 a A	17.6 ab AB	31.3 ab A	30.2 a A	1.83 a A	16.7 a AB	29.9 a A
	S3	36.6 a B	2.22 a A	16.6 b B	33.0 a A	29.7 ab B	1.89 a A	15.9 a AB	29.3 a A
N3	S0	37.9 a A	1.64 b A	23.2 a A	23.7 b A	30.3 a A	1.42 b A	21.6 a A	18.2 c A
	S1	39.6 a A	1.85 ab A	21.7 a A	28.4 ab A	31.6 a A	1.59 ab A	20.0 a A	23.1 bc A
	S2	40.7 a A	2.01 a AB	20.6 a A	33.7 a A	32.7 a A	1.72 a A	19.3 a A	28.7 a A
	S3	40.5 a A	2.04 a AB	20.0 a A	32.9 a A	33.2 a A	1.87 a A	18.1 a A	27.4 ab A
ANOVA									
N		**	**	**	**	**	*	**	**
S		*	**	**	**	*	**	*	**
N × S		*ns*	*ns*	*ns*	*ns*	*ns*	*ns*	*ns*	*ns*

The data are the means of four replicates (*N* = 4). Different lowercase letters after the data indicate the significant differences between the different S rates at the same N level (*P* < 0.05), and different capital letters after the data indicate the significant differences between the different N rates at the same S level (*P* < 0.05). The variance analysis used two-way ANOVA (**P < 0.01, *P < 0.05, *ns P* > 0.05). *N*_*c*_, nitrogen concentration in leaves; *S*_*c*_, sulfur concentration in leaves; Pn, net photosynthetic rate.

### Grain Yield of Maize

**FIGURE 2:**
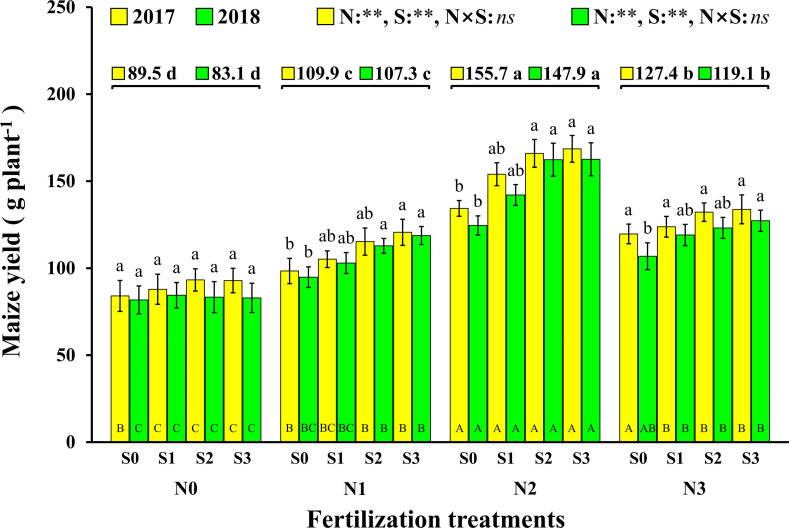
Effect of N and S on maize yield. The data are the means of four replicates, and the error bars represent the standard deviations. The lowercase letters above the bars indicate the significant differences between the different S rates at the same N level in the same year, and the different uppercase letters in the bars indicate the significant differences between the different N rates at the same S level in the same year. The number on the horizontal line above each group of bars indicates the average value of grain yield of maize at different N levels in the same year, and the lowercase letters after the numbers indicate the significant difference in yield at different N levels. The variance analysis used two-way ANOVA (^∗∗^*P* < 0.01, *ns P* > 0.05).

The change trend in maize yield in 2017 and 2018 was same, and the results show that both N and S had significant effects on maize yield, but there were no significant interactions between the two elements (Figure 2). The effect of N on maize yield was analyzed, and it is demonstrated that the maize yield with N application was significantly higher than that without N application (N0). Compared to N0 (89.5 g plant^–1^ and 83.1 g plant^–1^, 2017 and 2018), the maize yield with the N1, N2, and N3 levels increased by 22.8% and 29.0% (2017 and 2018), 74.0% and 78.0% (2017 and 2018), and 42.3% and 43.3% (2017 and 2018), respectively. The maize yield of S application was also significantly higher than that without S application (S0). Compared to S0 (109.1 g plant^–1^ and 94.5 g plant^–1^, 2017 and 2018), the maize yields with the S1, S2, and S3 levels increased by 7.9% and 9.4% (2017 and 2018), 16.1% and 20.8% (2017 and 2018), and 18.1% and 22.8% (2017 and 2018), respectively. At the N1 and N2 levels, maize yield increased with S application rates. For each S level, maize yield increased with N application rates and reached a maximum at the N2 level and then decreased at the N3 level. The highest yields were observed under the N2S2 and N2S3 treatments with yields of 166.0 g plant^–1^ and 162.3 g plant^–1^ (2017 and 2018), 168.6 g plant^–1^ and 162.6 g plant^–1^ (2017 and 2018), respectively.

### Grain Protein Concentration of Maize

**FIGURE 3:**
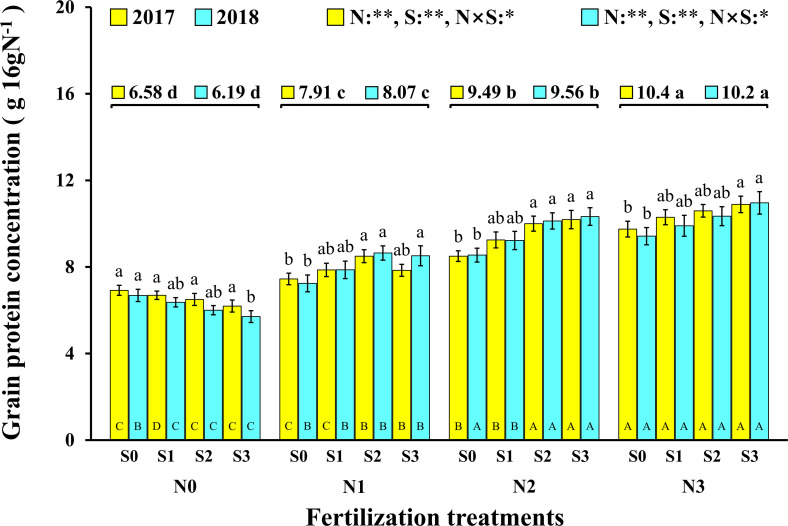
Effect of N and S on grain protein concentration of maize. The data are the means of four replicates, and the error bars represent the standard deviation. The lowercase letters above the bars indicate the significant differences between the different S rates at the same N level in the same year, and the different uppercase letters in the bars indicate the significant differences between the different N rates at the same S level in the same year. The number on the horizontal line above each group of bars indicates the average value of protein concentration of maize at different N levels in the same year, and the lowercase letters after the numbers indicate the significant difference in protein concentration at different N levels. The variance analysis used two-way ANOVA (^∗∗^*P* < 0.01, ^∗^*P* < 0.05).

In this experiment, the change trend in the grain protein concentration was the same in the two consecutive years (Figure 3). In 2017 and 2018, both N and S had significant effects on the grain protein concentration of maize, and their interaction reached a significant level. Correspondingly, the concentration of N and S in leaves and maize grains during the maturity stage (2018) increased significantly with the application of N and S, and there were significant interactions between N and S on grain N concentration (Table 2). For each S level, grain protein concentration in maize continuously increased with N application rates. The grain protein concentration in maize decreased as S application rates increased at the N0 level, which may be ascribed to the S application inhibiting the N absorption of maize at a low N supply. Under N application conditions, grain protein concentration was directly proportional to S application rates and reached a maximum (10.9 g 16gN^–1^, 11.0 g 16gN^–1^, 2017 and 2018) at the N3S3 treatment. It is worth emphasizing that the N2S3 treatment achieved the highest maize yield, but its grain protein concentration still reached a high level (10.2 g 16gN^–1^, 10.3 g 16gN^–1^, 2017 and 2018) and had no significant difference from the N3S3 treatment although it was significantly higher than the N0S0 treatment (6.9 g 16gN^–1^, 6.7 g 16gN^–1^, 2017 and 2018).

**TABLE 2:** N and S stoichiometry of maize leaves and grains during maturity stage (140 DAP).

Treatment		Leaf			Grain		
		*N*_*c*_	*S*_*c*_	N:S	*N*_*c*_	*S*_*c*_	N:S
		
		(mg g^–1^)	(mg g^–1^)		(mg g^–1^)	(mg g^–1^)	
N0	S0	14.6 a D	1.27 b A	11.6 a C	10.7 a B	0.89 b A	12.4 a A
	S1	14.7 a D	1.36 ab A	10.9 a B	10.2 ab C	0.99 ab A	11.1 ab A
	S2	13.9 ab D	1.45 ab A	9.66 ab C	9.60 ab C	1.18 ab A	8.29 b C
	S3	12.8 b D	1.54 a A	8.39 b C	9.13 b C	1.27 a A	7.31 b B
N1	S0	19.1 a C	1.29 b A	15.0 a B	11.6 b B	0.89 c A	13.4 a A
	S1	20.5 a C	1.41 ab A	14.6 a A	12.6 ab B	1.12 b A	11.4 ab A
	S2	20.4 a C	1.52 a A	13.5 ab B	13.8 a B	1.32 ab A	10.6 ab BC
	S3	18.9 a C	1.58 a A	12.0 b B	13.6 a B	1.43 a A	9.60 b BC
N2	S0	22.5 a B	1.31 b A	17.3 a AB	13.7 b A	0.97 b A	14.2 a A
	S1	23.8 a B	1.48 ab A	16.3 ab A	14.8 ab A	1.14 b A	13.3 ab A
	S2	24.2 a B	1.60 a A	15.3 ab AB	16.2 a A	1.38 a A	11.9 ab AB
	S3	23.5 a B	1.68 a A	14.2 b A	16.5 a A	1.50 a A	11.1 b AB
N3	S0	25.7 a A	1.40 b A	18.5 a A	15.1 b A	0.96 c A	15.8 a A
	S1	26.7 a A	1.57 ab A	17.1 ab A	15.8 ab A	1.10 bc A	14.4 ab A
	S2	27.1 a A	1.67 ab A	16.4 ab A	16.6 ab A	1.17 b A	14.2 ab A
	S3	26.7 a A	1.77 a A	15.2 b A	17.5 a A	1.34 a A	13.2 b A
ANOVA							
N		**	**	**	**	*	**
S		*	**	**	*	**	**
N × S		*ns*	*ns*	*ns*	*	*ns*	*ns*

The data are the means of four replicates (*N* = 4). Different lowercase letters after the data indicate the significant differences between the different S rates at the same N level (*P* < 0.05), and different capital letters after the data indicate the significant differences between the different N rates at the same S level (*P* < 0.05). The variance analysis used two-way ANOVA, (**P < 0.01, *P < 0.05, *ns P* > 0.05). *N*_*c*_, nitrogen concentration of samples; *S*_*c*_, sulfur concentration of samples.

### Grain Ultrastructure of Maize

**FIGURE 4:**
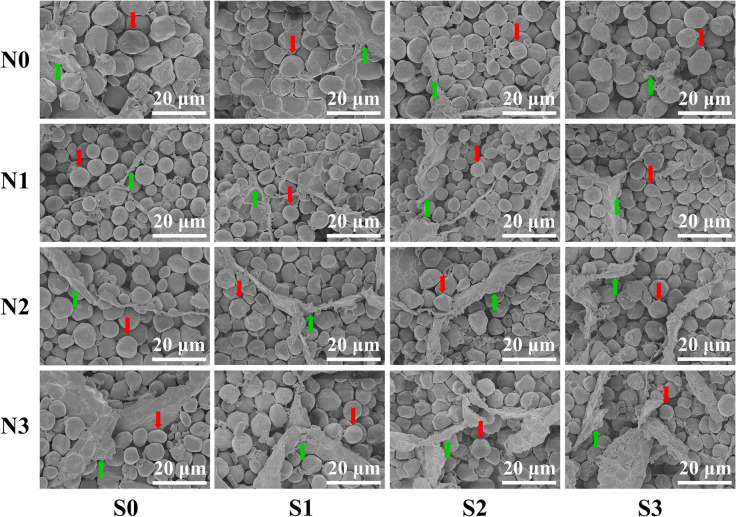
Grain endosperm ultrastructure of maize at different N and S rates. The weight of individual grains was calculated based on the grain weight per hundred kernels of the maize, and the grains equal to the average grain weight were selected by weighing with an electronic balance. The selected samples of maize grains were sliced with a blade (thickness ≈ 1 mm). The prepared samples were observed and photographed using a scanning electron microscope. In this image, the red arrows indicate the starch granules, the green arrows indicate the matrix proteins, and the bars represent 20 μm.

To verify the regularity of the changes in protein concentration in grains, the ultrastructure of maize grains was tested. An SEM image showed that the change trend of the matrix proteins was completely consistent with the measured value of protein concentration in grains (Figure 4). At the N0 level, the starch granules in the maize grains were larger and irregularly shaped, and their arrangement was loose with a small number of matrix proteins interspersed in the gaps of the starch granules. At the N1 level, the starch granules in maize grains were small and spheroidal, which were arranged closely and orderly and had a large amount, and the divided matrix proteins were interspersed in the gaps of the starch granules. At the N2 and N3 levels, the starch granules in maize grains were closely arranged, and a large number of matrix proteins was interspersed in the gaps of the starch granules. In terms of S application, the matrix proteins in maize grains at other N levels increased as S application rates increased except for the N0 level.

### Grain Amino Acids Analysis of Maize

**FIGURE 5:**
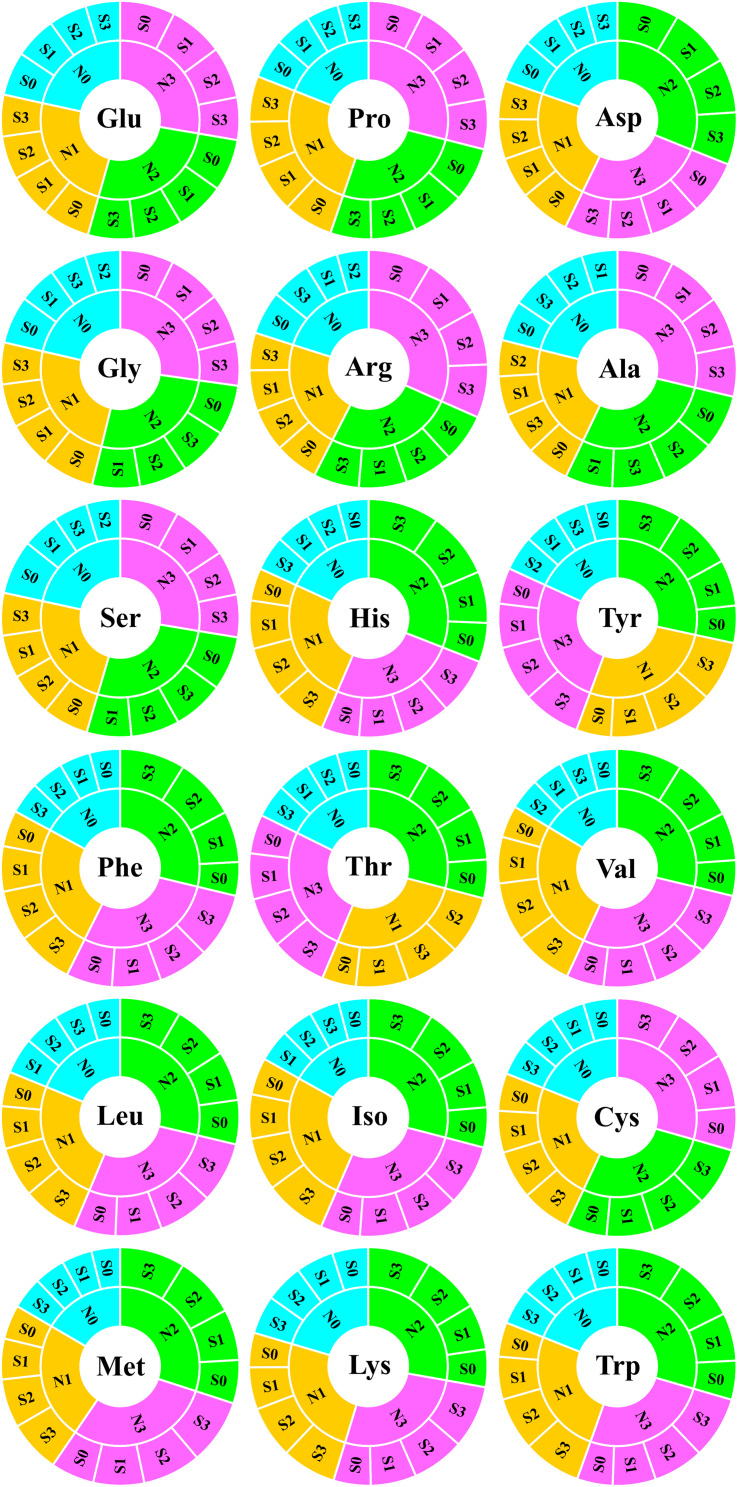
Analysis of amino acids in maize grains. In this image, the abbreviations at the center of the circle represent the amino acid type. The fan-shaped regions of different colors indicate the amino acid concentration of grains at different N rates, and each grid surrounding the outer ring of the same color region indicates the amino acid concentration of different S rates at the same N level. Both the fan-shaped area and the outer ring grid are arranged in descending clockwise order according to the area size. Glu, glutamate; Pro, proline; Asp, aspartic acid; Gly, glycine; Arg, arginine; Ala, alanine; Ser, serine; His, histidine; Tyr, tyrosine; Phe, phenylalanine; Thr, threonine; Val, valine; Leu, leucine; Iso, isoleucine; Cys, cysteine; Met, methionine; Lys, lysine; Trp, tryptophan.

**FIGURE 6:**
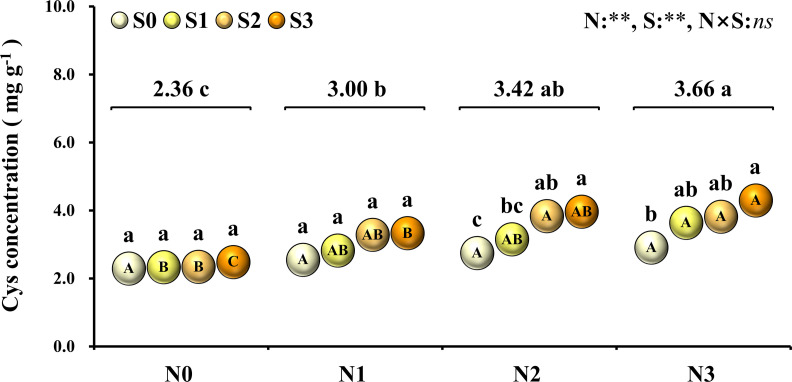
Concentration of cysteine in maize grains at different N and S rates. The data are the means of four replicates. The lowercase letters above the spheres indicate the significant differences between the different S rates at the same N level, and the different uppercase letters in the spheres indicate the significant differences between the different N rates at the same S level. The number on the horizontal line above each group of spheres indicates the average value of concentration of cysteine at different N levels, and the lowercase letters after the numbers indicate the significant difference in concentration of cysteine at different N levels. The variance analysis used two-way ANOVA (^∗∗^*P* < 0.01, *ns P* > 0.05).

To evaluate the nutritional quality of maize grains, the concentration of amino acids in maize grains was measured and analyzed in 2018 (Figure 5). At the N0 level, various amino acids in maize grains were at their lowest level. At the N1 level, except for tyrosine and threonine, the concentration of other amino acids was at a lower level and slightly higher than that of the N0 treatment. At the N2 level, the concentrations of aspartic acid, histidine, tyrosine, phenylalanine, threonine, valine, leucine, isoleucine, methionine, lysine, and tryptophan were highest, and among these amino acids, other amino acids were EAA except for aspartic acid. At the N3 level, the concentration of glutamate, proline, glycine, arginine, alanine, serine, and cysteine were highest, and among these amino acids, other amino acids were NAA except for cysteine. In addition to the N0 level, the various NAA concentration of S application at other N levels was lower than the S0 level, but at each N level, the various EAA concentration of S application was higher than S0 level. In particular, as a sulfur-containing amino acid, the concentration of cysteine in maize grains increased with N application rates. At the N2 and N3 levels, the concentration of cysteine in maize grains increased with the S application rate (Figure 6).

**FIGURE 7:**
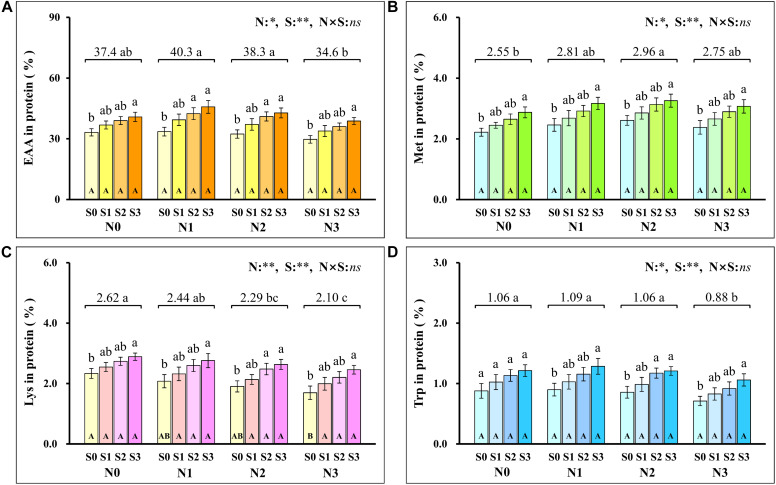
The proportion of total essential amino acids **(A)**, methionine **(B)**, lysine **(C)**, tryptophan **(D)** in protein at different N and S rates. The data are the means of four replicates, and the error bars represent the standard deviation. The lowercase letters above the bars indicate the significant differences between the different S rates at the same N level, and the different uppercase letters in the bars indicate the significant differences between the different N rates at the same S level. The number on the horizontal line above each group of bars indicates the average value of proportion of amino acids in protein at different N levels, and the lowercase letters after the numbers indicate the significant difference in proportion of amino acids in protein at different N levels. The variance analysis used two-way ANOVA (^∗∗^*P* < 0.01, ^∗^*P* < 0.05, *ns P* > 0.05). EAA, total essential amino acids; Met, methionine; Lys, lysine; Trp, tryptophan.

Analysis of the proportion of amino acids in grain protein showed that the total EAA (Figure 7A) in grain protein increased first and then decreased with the increase of N application rates and reached the minimum (34.6%) at the N3 level. At each N level, the total EAA in grain protein increased with S application rates and reached the maximum (45.8%) at N1S3 treatment. For each S level, the total EAA in grain protein had no significant difference at each N application rate. Notably, the highest maize yield was obtained at the N2S3 treatment, but its proportion (42.8%) of EAA in grain protein was not significantly different from the N1S3 treatment.

To further evaluate the balance of amino acids in grain protein, the changes in several amino acids that were usually deficient in maize grains were analyzed emphatically. Methionine (Figure 7B) in grain protein increased with N application rates and reached a maximum (2.96%) at the N2 level. At each N level, methionine in protein increased as S application rates increased and reached the maximum (3.26%) at the N2S3 treatment. For each S level, methionine in protein had no significant difference at each N application rate. The lysine (Figure 7C) in protein continuously decreased as N application rates increased, and the minimum (2.10%) was observed at the N3 level. At the S0 level, lysine in protein decreased with N application rates and reached a minimum (1.70%) at the N3 level. At each N level, lysine in protein increased with S application rates, and the maximum (2.89%) was observed at the N1S3 treatment. Except for the S0 level, there was no significant difference in lysine with N application rates at other S levels. Compared with the N0 level, a small amount (N1 level) and a proper amount (N2 level) of N application had no significant effect on tryptophan (Figure 7D) in grain protein, but excessive N application (N3 level) significantly reduced the concentration of tryptophan in grain protein. Except for the N0 level, tryptophan in protein at other N levels increased as S application rates increased and reached the maximum (1.28%) at the N1S3 treatment. For each S level, tryptophan in grain protein had no significant difference at each N application rate.

## Discussion

### Regulation of N and S to Increase Maize Yield by Enhancing Photosynthesis

Photosynthesis is the biochemical basis of the synthesis of photosynthates, such as sucrose and starch, which directly determine the crop yield (Lunn and Hatch, 1995; Kruger and Volin, 2006). Enhancing photosynthesis is an important guarantee for high crop yield (Lawson et al., 2012). In this study, coordinated application of N and S significantly enhanced GSH content (Figures 1E,F), Rubisco activity (Figures 1C,D), and photosynthetic rate (Figures 1A,B) of maize leaves, which was consistent with the change in maize yield. The increase in photosynthetic rate may benefit from the removal of excessively accumulated H_2_O_2_ (Figures 1G,H) in leaves by GSH to reduce oxidative damage to the Rubisco, which can be inferred from the result that the Rubisco activity was directly proportional to the GSH content, and it was inversely proportional to H_2_O_2_ content. Actually, plants inevitably produce reactive oxygen species (ROS), such as H_2_O_2_ during the photosynthesis processes (Foyer, 2018; Smirnoff and Arnaud, 2019). An ROS, such as H_2_O_2_, has a dual role in plant biology; it is a key regulator of plant growth, development, and defense pathways, and it is a toxic by-product of aerobic metabolism (Mittler et al., 2004; Saxena et al., 2016; Exposito-Rodriguez et al., 2017). H_2_O_2_, as one of the most stable ROS in plants, can attack Rubisco and cause severe oxidative damage. In this study, Rubisco activity decreased with increasing H_2_O_2_ content, and the decrease in Rubisco activity directly led to a decrease in photosynthetic rate, which was consistent with previous reports (González-Moro et al., 1997; Li et al., 2004). The redox imbalance in the thiol-disulfide network was ascribed to increased generation of ROS, and GSH can counteract the accumulation of ROS, such as H_2_O_2_ (König et al., 2018). In this study, the H_2_O_2_ content decreased with the increase of GSH content, and the photosynthetic rate increased with GSH content, which was consistent with reported results (Fatma et al., 2014). In this study, the GSH content in maize leaves can be achieved by adjusting the application of N and S, which has been confirmed in relevant studies (Gutiérrez-Gamboa et al., 2016; Liang et al., 2016). Therefore, regulating the redox balance of maize leaves by coordinating the supply of N and S nutrients to control the GSH content is an important method for improving photosynthesis. In addition, N and S may also improve photosynthesis by increasing leaf area and chlorophyll concentration of maize, thereby increasing grain yield (Li et al., 2019).

### Regulation of N and S to Optimize Grain Protein Concentration and Amino Acid Balance by Increasing Cysteine in Maize Grains

**FIGURE 8:**
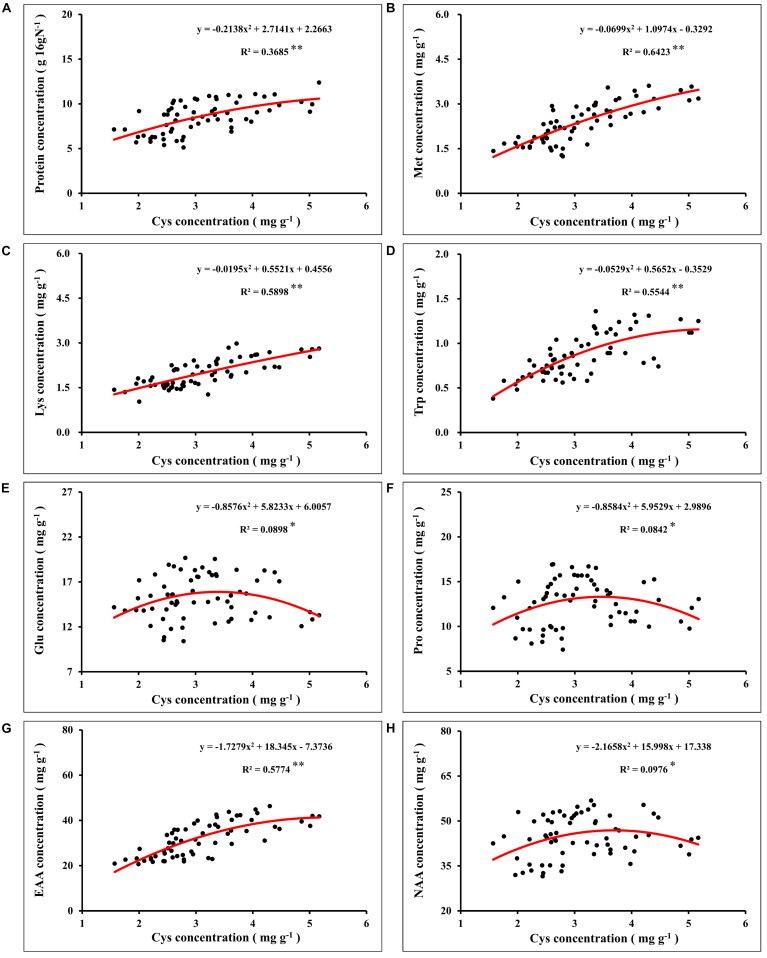
Correlation analysis between the concentration of grain protein **(A)**, methionine **(B)**, lysine **(C)**, tryptophan **(D)**, glutamate **(E)**, proline **(F)**, total essential amino acids **(G)**, total non-essential amino acids **(H)**, and cysteine concentration in maize grains. In this image, the red curve shows the correlation between the concentration of protein, concentration of corresponding amino acids, and cysteine concentration in maize grains (*n* = 64). Met, methionine; Lys, lysine; Trp, tryptophan; Glu, glutamate; Pro, proline; EAA, total essential amino acids; NAA, total non-essential amino acids.

In global maize production, grain protein concentration has shown a downward trend with the increase of grain yield (Duvick and Cassman, 1999; Ciampitti and Vyn, 2012). Nutrient management was an important strategy to simultaneously increase maize yield and grain protein concentration (Zhang et al., 2020). In this study, synergistic application of N and S simultaneously increased maize yield and grain protein concentration, which might have benefited from the mutual promotion of N and S accumulation in grains. The analysis of N and S concentrations in maize leaves during the growth stage (Table 1) and maturity stage (Table 2) showed that the proper N application (N2 level) promoted the absorption of S. Similarly, suitable S application (S2 level) increased the N accumulation in leaves, which is consistent with the previous report (Li et al., 2019). During the maturity stage, signs of mutual promotion of absorption of N and S were also observed in maize grains (Table 2). Grain protein of maize was the main compound that stored N and S, which accumulated in the protein as the form of amino acids (Sriperm et al., 2010). Analysis of the amino acids in maize grains showed that the concentration of various amino acids significantly increased with N application (Figure 5). When N supply was sufficient, as a sulfur-containing amino acid, cysteine concentration further increased with the coordinated application of S fertilizer. Cysteine is the basis for the formation of protein disulfide bonds, and the formation of disulfide bonds enhances protein stability (Depuydt et al., 2011). Limited by the conformation of protein, the increase of cysteine concentration provided more possible sites and opportunities for the formation of disulfide bonds, which contributed to the formation and improved stability of protein (Hogg, 2003; Liu et al., 2016). The results showed that protein concentration of grains correlated significantly with cysteine concentration in maize (Figure 8A), which indicated that the formation and stability of protein were enhanced with cysteine concentration. Thus, the sufficient formation of disulfide bonds may be the main reason for synergistic application of N and S to increase the protein concentration of maize grains.

Nitrogen supply played a dominant role in determining the quality of storage protein in seeds, and S application could regulate the composition of seed protein under the determined N supply level (Tabe et al., 2002; Li et al., 2016). In this study, the synergistic application of N and S significantly affected the concentration of various amino acids in the protein (Figure 5). According to Salamini and Soavc (1982), grain protein of maize was classified as albumins (3%), globulins (3%), glutelins (34%), and zeins (60%). As the most abundant storage protein in maize endosperm, zeins were high in NAA, such as glutamine, proline, and alanine, and severely lacked EAA, such as lysine, tryptophan, and methionine, which limited the nutritional quality of maize (Coleman and Larkins, 1999). Based on the difference in solubility, zeins were divided into α-, β-, γ-, and δ-zeins (Esen, 1987). Among these types of zeins, γ-zeins are a rich source of cysteine, and δ-zeins contain a high proportion of methionine (Kirihara et al., 1988; Swarup et al., 1995). The β-zeins are high in two sulfur-containing amino acids, cysteine and methionine (Randall et al., 2004). The α-zeins account for more than 70% of the total zeins, but lack lysine and cysteine, and the methionine concentration is poor, which seriously damaged the balance of amino acids in maize grains (Wu et al., 2012). Reducing the α-zein concentration can lead to a compensatory increase of the concentration of β-, γ-, and δ-zein and non-zein protein (Landry, 2002). In this study, under sufficient N supply, the cysteine concentration was increased with S application (Figure 6), and the proportion of lysine (Figure 7C), tryptophan (Figure 7D), and total EAA (Figure 7A) in grain protein was also significantly increased. Simultaneously, coordinated application of N and S reduced the NAA concentration, such as glutamic acid and proline, under the premise of ensuring a steady increase of protein concentration, which might benefit from the increase in cysteine regulated by S application, thereby inhibiting the concentration of α-zeins and causing the compensatory increase of EAA-rich non-zein protein. During the development of maize grains, cysteine residues in β- and γ-zeins cross-linked with each other and with other amino acids (Gibbon and Larkins, 2005). However, α-zeins were devoid of cysteine, which reduced the possibility of participation in protein formation, thus providing more opportunities for EAA, such as methionine, lysine, and tryptophan, into proteins. In this study, the concentrations of methionine (Figure 8B), lysine (Figure 8C), tryptophan (Figure 8D), and EAA (Figure 8G) continuously increased with cysteine concentration, and the concentrations of glutamic acid (Figure 8E), proline (Figure 8F), and NAA (Figure 8H) increased first and then decreased with cysteine concentration. These relationships indicated that protein formation preferentially accumulated EAA and inhibited NAA at a high enough cysteine concentration, thereby optimizing amino acid composition of protein and achieving amino acid balance.

### Stoichiometry of N and S in Synergistically Improving Grain Yield, Grain Protein Concentration, and Quality of Maize

**FIGURE 9:**
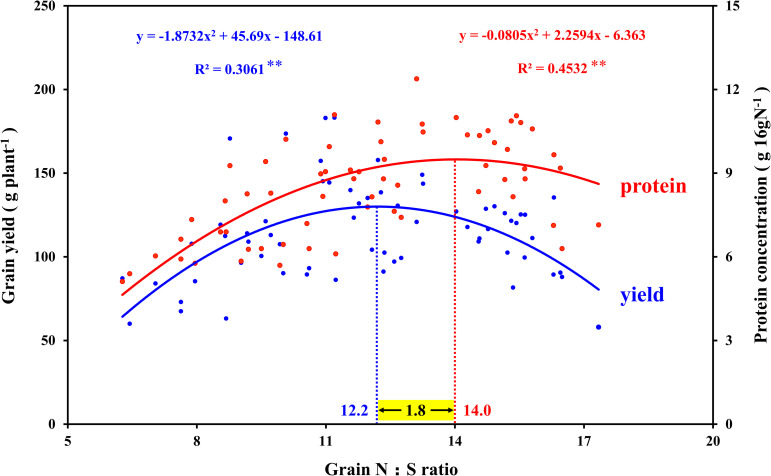
Correlation analysis between the grain yield, grain protein concentration, and N:S ratio in maize grains. In this image, the blue curve shows the correlation between grain yield and N:S ratio in maize grains (*n* = 64), and the intersection of the blue dotted line and the blue curve indicates the maximum value of the curve. The red curve shows the correlation between grain protein concentration and N:S ratio in maize grains (*n* = 64), and the intersection of the red dotted line and the red curve indicates the maximum value of the curve.

In view of the interaction between crop nutrients, stoichiometry is often used to quantify the interaction between nutrients and determine the nutrients level of crops (Divito et al., 2016; Salvagiotti et al., 2017). The analysis of N and S concentration in maize plants showed good performance in predicting grain yield, which was considered to be an advantageous tool for N and S regulation in crops (Pagani and Echeverría, 2011). In this study, the N:S ratio of leaves that obtained the maximum photosynthetic rate (N2S3 and N2S2 treatments) were 16.6 (silking stage) and 16.7 (grain-filling stage), respectively (Table 1). The N:S ratio of the leaves at the maturity stage decreased in each treatment, which was ascribed to the N export from the leaf being faster than the S export (Table 2). At the maturity stage, the N:S ratio of the leaves of the N2S2 and N2S3 treatments that achieved the highest yield were 15.3 and 14.2, respectively. Analogous research showed that the range of the leaves’ N:S ratio suitable for maize growth was 14.4–18.7, which is consistent with our results (Li et al., 2019). The correlation analysis between the maize yield, grain protein concentration, and N:S ratio of grains showed that maize yield reached a maximum at 12.2 (N:S ratio), and grain protein concentration reached a maximum at 14.0 (N:S ratio). Obviously, the optimal N:S ratio of yield and grain protein concentration did not coincide (Figure 9), which needs to be further analyzed. A lower N:S ratio indicated sufficient or excessive S supply, but it may also be ascribed to N deficiency. Conversely, a higher N:S ratio meant an excessive N supply or an S deficiency (Blake-Kalff et al., 2000). In this study, at the highest S application level (S3 level), the photosynthetic rate of maize leaves, maize yield, and grain protein concentration all achieved the maximum, which indicated that S application was sufficient rather than excessive. Therefore, the lower N:S ratio was ascribed to N deficiency. At the maximum N application level (N3 level), maize yield significantly decreased, which indicated that this N application level has led to an oversupply of N. Thus, the higher N:S ratio was due to the excessive supply of N. In addition, the super-high grain N:S ratio appeared in the experimental treatment of excessive N application without S application (N3S0 treatment). This fertilization method (N3S0 treatment) not only led to low S concentration in maize grains, but also low S concentration in maize leaves, which severely limited the photosynthetic rate of the leaves. The lower photosynthesis rate led to poor grain filling, so the maize yield was lower. When the N:S ratio of grains was at 12.2, it meant that the optimal ratio of N and S concentration in grains was obtained, and thereby, the maximum maize yield was obtained. When the N:S ratio of grains was at 12.2–14.0, it indicated excessive supply of N, and the grain protein concentration increased with N application, which is consistent with previous studies (Hou et al., 2012; Qiu et al., 2015). When the N:S ratio of grain exceeded 14.0, it indicated the deficiency of S, and the grain protein concentration also decreased with S deficiency. However, when maize yield was at the highest level, the grain protein concentration did not decrease significantly, and the total protein accumulation (maize yield × grain protein concentration) reached the maximum value (16.8 g plant^–1^). Therefore, the most efficient method to obtain the maximum maize yield without sacrificing grain protein concentration was to precisely regulate the N:S ratio of grains.

**FIGURE 10:**
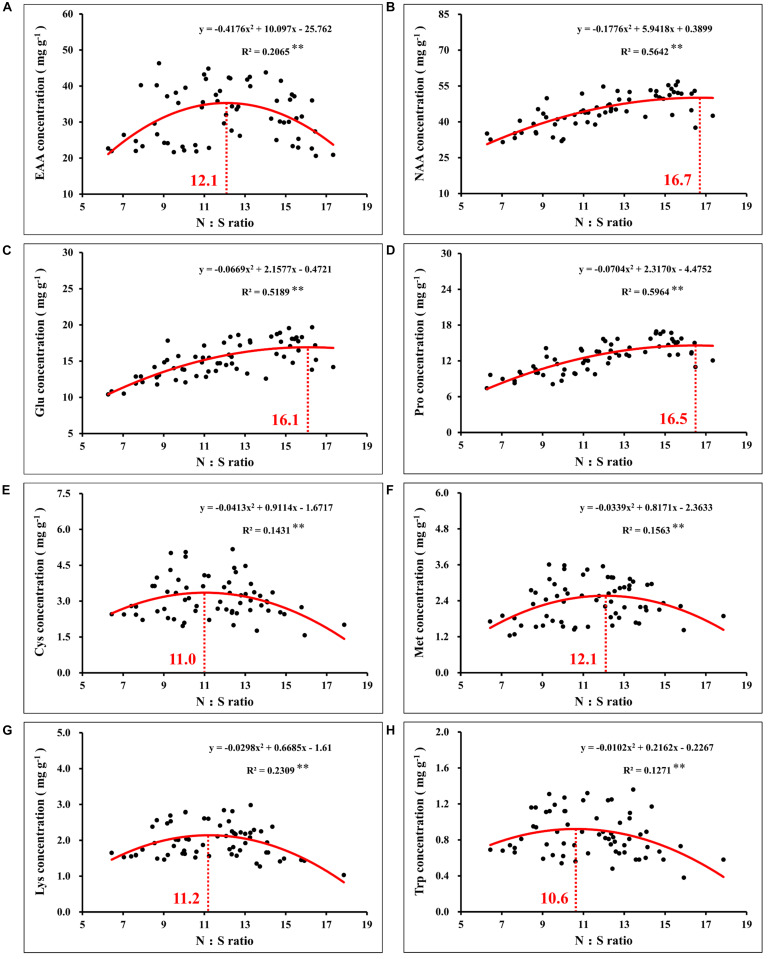
Correlation analysis between the concentration of total essential amino acids **(A)**, total non-essential amino acids **(B)**, glutamate **(C)**, proline **(D)**, cysteine **(E)**, methionine **(F)**, lysine **(G)**, tryptophan **(H)**, and N:S ratio in maize grains. In this image, the red curve shows the correlation between the concentration of amino acids and N:S ratio in maize grains (*n* = 64), and the intersection of the red dotted line and the red curve indicates the maximum value of the curve. EAA, total essential amino acids; NAA, total non-essential amino acids; Glu, glutamate; Pro, proline; Cys, cysteine; Met, methionine; Lys, lysine; Trp, tryptophan.

Notably, correlation analysis between the amino acids and N:S ratio in grains showed that the optimal N:S ratio of EAA (Figure 10A), such as cysteine (Figure 10E), methionine (Figure 10F), lysine (Figure 10G), and tryptophan (Figure 10H), were all at a lower level (10.6–12.1), and the optimal N:S ratio values of NAA (Figure 10B), such as glutamic acid (Figure 10C) and proline (Figure 10D), were higher (16.1–16.7). Thus, in order to obtain higher nutritional quality of grains, the grain N:S ratio should be controlled at about 11.0–12.0. According to the respective experimental treatments, N2S2 and N2S3 treatments achieved the highest grain yield, and grain protein concentration was also at a high level. The N:S ratio of the N2S2 and N2S3 treatments were 11.9 and 11.1, respectively (Table 2), which was highly consistent with the range of grain N:S ratio that achieved the highest nutritional quality. The grain N:S ratio affected maize yield, grain protein concentration, and various amino acid concentration. The maize yield and nutritional quality were simultaneously improved at the suitable range (11.0–12.0) of grain N:S ratio.

**FIGURE 11:**
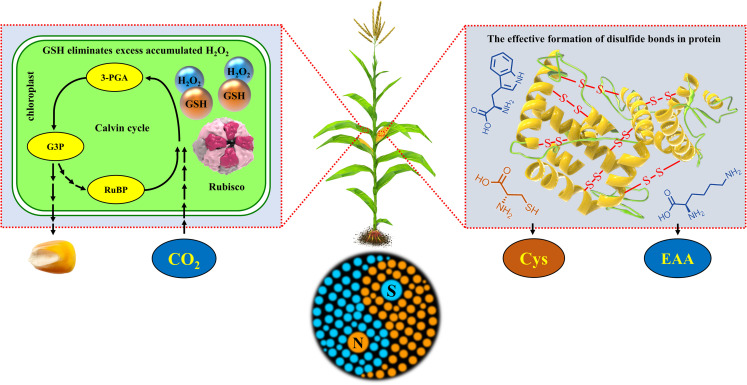
Schematic diagram of the synergistic mechanism of N and S synergistically improving maize yield, grain protein concentration, and quality. The box on the left represents the physiological response during the Calvin cycle in the chloroplast of maize leaves, and the box on the right represents the synthesis and accumulation of protein in maize grains. RuBP, ribulose-1,5-bisphosphate; 3-PGA, 3-phosphoglycerate acid; G3P, glyceraldehyde-3-phosphate; Rubisco, ribulose-1,5-bisphosphate carboxylase/oxygenase; GSH, glutathione; H_2_O_2_, hydrogen peroxide; Cys, cysteine; EAA, essential amino acids (methionine, lysine, tryptophan, etc.); -S- S-, disulfide bonds in protein.

Overall, the synergetic regulation of N and S simultaneously improved the yield and nutritional quality of maize by regulating the redox balance of leaves and balance of amino acids in grains (Figure 11).

## Conclusion

Coordinated application of N and S significantly affected maize growth, yield, and nutritional quality. The GSH in the maize leaves increased the photosynthetic rate by maintaining the redox balance, thereby increasing maize yield. The cysteine in grains optimized the concentration of grain protein and balance of amino acids by regulating the ratio of amino acids. The coordinated regulation of N and S synergistically improved the yield and nutritional quality of maize, which met the requirement for sustainable development in maize production and provided a new theoretical basis and method for the high-yield and high-quality production of maize.

## Data Availability Statement

The raw data supporting the conclusions of this article will be made available by the authors, without undue reservation.

## Author Contributions

QG and GM conducted an overall design for this study. SL completed the experiments and wrote the manuscript with guidance from QG and YW. SC, XZ, and GM provided suggestions and edited the manuscript. All authors contributed to the article and approved the submitted version.

## Conflict of Interest

The authors declare that the research was conducted in the absence of any commercial or financial relationships that could be construed as a potential conflict of interest.
